# Enterovirus 71 Represses Interleukin Enhancer-Binding Factor 2 Production and Nucleus Translocation to Antagonize ILF2 Antiviral Effects

**DOI:** 10.3390/v12010022

**Published:** 2019-12-23

**Authors:** Jing Jin, Wenbiao Wang, Sha Ai, Weiyong Liu, Yu Song, Zhen Luo, Qi Zhang, Kailang Wu, Yingle Liu, Jianguo Wu

**Affiliations:** 1 State Key Laboratory of Virology, College of Life Sciences, Wuhan University, Wuhan 430072, China; jinjing47@126.com (J.J.); 2016202040036@whu.edu.cn (S.A.); wyliu@hust.edu.cn (W.L.); songyu@whu.edu.cn (Y.S.); gracetey@whu.edu.cn (Q.Z.); wukailang@whu.edu.cn (K.W.); mvlwu@whu.edu.cn (Y.L.); 2 Guangdong Key Laboratory of Virology, Institute of Medical Microbiology, Jinan University, Guangzhou 510632, China; shabiao1212@whu.edu.cn (W.W.); zhluo18@jnu.edu.cn (Z.L.)

**Keywords:** enterovirus 71, EV71, EV71 nonstructural protein 2B, interleukin enhancer-binding factor 2, ILF2, virus infection, virus replication

## Abstract

Enterovirus 71 (EV71) infection causes hand-foot-mouth disease (HFMD), meningoencephalitis, neonatal sepsis, and even fatal encephalitis in children, thereby presenting a serious risk to public health. It is important to determine the mechanisms underlying the regulation of EV71 infection. In this study, we initially show that the interleukin enhancer-binding factor 2 (ILF2) reduces EV71 50% tissue culture infective dose (TCID50) and attenuates EV71 plaque-formation unit (PFU), thereby repressing EV71 infection. Microarray data analyses show that ILF2 mRNA is reduced upon EV71 infection. Cellular studies indicate that EV71 infection represses ILF2 mRNA expression and protein production in human leukemic monocytes (THP-1) -differentiated macrophages and human rhabdomyosarcoma (RD) cells. In addition, EV71 nonstructural protein 2B interacts with ILF2 in human embryonic kidney (HEK293T) cells. Interestingly, in the presence of EV71 2B, ILF2 is translocated from the nucleus to the cytoplasm, and it colocalizes with 2B in the cytoplasm. Therefore, we present a distinct mechanism by which EV71 antagonizes ILF2-mediated antiviral effects by inhibiting ILF2 expression and promoting ILF2 translocation from the nucleus to the cytoplasm through its 2B protein.

## 1. Introduction

Enterovirus 71 (EV71), a positive-stranded RNA virus, is a member of the Enterovirus genus within the family Picornaviridae [1,2]. EV71 infection may cause hand-foot-mouth disease (HFMD), meningoencephalitis, neonatal sepsis, and fatal encephalitis in children [3,4]. EV71 has a signal-stranded, positive-sense RNA genome with one open reading frame that encodes a polyprotein of approximately 250 kDa carrying three regions named P1, P2, and P3 [5]. Upon EV71 infection, the protein precursor is cleaved into four structural proteins (VP1, VP2, VP3, and VP4) and seven nonstructural proteins (2A, 2B, 2C, 3A, 3B, 3C, and 3D) [6]. Among the proteins, 2B is involved in EV71 RNA replication [7], localizes in the mitochondria to activate the mitochondrial cell death [8], enhances viral release [9], and antagonizes retinoic acid-inducible gene I (RIG-I)-mediated antiviral effects [10]. 

Interleukin enhancer-binding factor 2 (ILF2) is a transcriptional activator which regulates interleukin 2 (IL-2) expression [11]. ILF2 together with its binding partner ILF3 regulates DNA replication [12,13], transcription [14], translation [15,16], mRNA splicing [17], and microRNA biogenesis [18,19,20]. ILF2 also plays a role in the progression of pancreatic carcinoma [21,22], hepatocellular carcinoma [20,23], lung cancer [24], esophageal squamous cell carcinoma [25], gastric cancer [26], and breast cancer [27]. Moreover, ILF2 regulates the replication of immunodeficiency virus type 1 [28], hepatitis C virus [29], rhinovirus type 2 [30], white spot syndrome virus [31], human papilloma virus [32], and respiratory syndrome virus [33]. However, the mechanisms underlying the control of ILF2-mediated antiviral effects have not been reported, and the role of ILF2 in EV71 replication is unknown.

In previous studies, we demonstrated that EV71 nonstructural protein 3D (also known as *RNA*-*dependent* *RNA* polymerase) binds to the NACHT, LRR, and PYD domain-containing protein 3 (NLRP3, the sensor component of NLRP3 inflammasome) to enhance inflammasome activation [34], and that ILF2 interacts with NLRP3 to inhibit inflammasome activation [35]. These results suggest that ILF2 may play roles in EV71 infection. In this study, we further determine the effect of ILF2 on EV71 infection. This is the first study showing that ILF2 reduces EV71 50% tissue culture infective dose (TCID50) and plaque-forming unit (PFU), providing evidence that ILF2 represses EV71 infection. In contrast, EV71 represses ILF2 mRNA expression and protein production. In addition, EV71 nonstructural protein 2B interacts with ILF2 to attenuate ILF2 nucleus translocation and promote the 2B-ILF2 colocalization in the cytoplasm. Therefore, we present a distinct mechanism by which EV71 antagonizes ILF2-mediated antiviral effects by inhibiting ILF2 expression and promoting ILF2 translocation from the nucleus to the cytoplasm through 2B protein.

## 2. Materials and Methods

### 2.1. Reagents

Phorbol-12-myristate-13-acetate (TPA) (#P8139), murine monoclonal HA antibody (H6908), antiglyceraldehyde-3-phosphate dehydrogenase (GAPDH) antibodies (#G9295), and carboxymethylcellulose (CMC-nZVI) (101839688) were purchased from Sigma (St. Louis, MO, USA,). RPMI 1640 medium, Dulbecco’s modified Eagle medium (DMEM), and fetal bovine serum (FBS) were purchased from Gibco (Grand Island, NY, USA). Anti-ILF2 was purchased from Santa Cruz Biotechnology (SC-365283) (Santa Cruz, CA, USA). Anti-EV71 3C antibody (#A10003) and Murine monoclonal green fluorescent protein (GFP) antibody (#AE012) were purchased from ABclonal Technology (Wuhan, China). Protease inhibitor cocktail (#04693132001) was purchased from Roche (Pleasanton, CA, USA). Protein markers (#26616) were purchased from Fermentas (Burlington, ON, Canada). Polyvinylidene fluoride (PVDF) membranes (#IPVH00010) were purchased from Millipore Corporation (Bedford, MA, USA). FITC-conjugated anti-mouse antibodies (#133702A) and Dylight 649-conjugated antirabbit secondary antibodies (#ATPSE2901) were purchased from Abbkine (San Diego, CA, USA). Bovine serum albumin (BSA) (#B0014K061000) was purchased from Biosharp (Hefei, China). 

### 2.2. Cell Lines

Human embryonic kidney (HEK293T) cells, African green monkey kidney epithelial (Vero) cells, human rhabdomyosarcoma (RD) cells, and human leukemic monocyte (THP-1) cells were purchased from American Tissue Culture Collection (Manassas, VA, USA). Cells were cultured in DMEM supplemented with 10% FBS, 100 U/mL penicillin, and 100 μg/mL streptomycin at 37 °C under 5% CO_2_.

### 2.3. Stimulation of THP-1 Cells and Differentiation into Adherent Macrophages

THP-1 cells were differentiated into macrophages under the stimulation of 60 nM TPA. TPA was removed after 12–16 h, and cells were cultured for additional 24 h.

### 2.4. Plasmid Construction

The EV71 genome fragments encoding 2B, 3C, 3A, 3C, and 3D proteins were cloned into pEGFPC1 between *Hind*III and *Sal*I sites, which resulted in the formation of GFP fusion protein. The ILF2 gene was cloned into pCaggs-HA between *Eco*RI and *Xho*I sites.

### 2.5. Lentivirus Construction

GFP protein on pLenti CMV GFP Puro vector (#17448) (Addgene, Watertown, MA, USA) was replaced with a 3 × FLAG sequence, and some stringent restriction sites (*Xba*I, *EcoR*V, *BstB*I, and *BamH*I) were added before the FLAG tag. The ILF2 gene was constructed in pLenti vector, and was then transfected into HEK293T cells with psPAX2 (#12260) (Addgene, Watertown, MA, USA) and PMD2.G (#12259) (Addgene, Watertown, MA, USA) plasmid via Lipo 2000 (52887) (Invitrogen, Carlsbad, CA, USA). The primers used were 5′-CTAGTCTAGAATGAGGGGTGACAGAGGCCG-3′ and 5′-GGAAGATCTCTCCTGAGTTTCCATGCTTTC-3′. The supernatants of transfected cells were collected after 36 h of incubation and used to infect RD cells with 4 μg/mL polybrene (#H9268) (Sigma). After 48 h of culture, the transduced cells were screened under the selection medium with 2.5 μg/mL puromycin (Sigma). Transgenic protein expression was analyzed by Western blotting.

### 2.6. Real-Time PCR 

For real-time PCR, the total RNA was isolated using TRIzol reagent (#15596018) (Invitrogen) and an Ultrapure RNA Kit (#CW0597) (CWBIO) in accordance with the manufacturer’s instructions. Genomic DNA was digested using DNase I. The complementary DNA was then synthesized using M-MLV Reverse Transcriptase (#M1705) (Promega) in accordance with the manufacturer’s instructions. Quantitative RT-PCR analysis was performed using the Roche LC480 and SYBR RT-PCR kits (DBI Bio-science, Ludwigshafen, Germany). Real-time PCR primers were designed by Primer Premier 5.0; their sequences were as follows: ILF2 forward, 5′-GATAATCTGATTGTGGCTCC-3′, ILF2 reverse, 5′-CGTTGGCAGAATCTTGAGTA-3′; GAPDH forward, 5′-AAGGCTGTGGGCAAGG-3′, GAPDH reverse, 5′-TGGAGGAGTGGGTGTCG-3′.

### 2.7. Western Blotting

RD cells were lysed using a lysis buffer of 50 mM Tris–HCl, pH 7.5, 300 mM NaCl, 1% Triton-X, 5 mM EDTA, and 10% glycerol, and protein was quantitated by the Bradford method (Bio-Rad, Hercules, CA). Quantified samples were run on 10 SDS-PAGE gels and transferred onto Immobilon-P Transfer Membranes (PVDF) (Millipore) using a transfer device. After blocking, PVDF membranes were incubated with primary antibodies overnight at 4 °C and then incubated with secondary antibodies at room temperature for 2 h. Blots were developed using a chromogenic solution on Chemiluminescence instrument (Fujifilm LAS-4000). 

### 2.8. 50% Tissue Culture Infective Dose (TCID50) Assay

Vero cells were grown in 96-well plates to 90% confluence and inoculated with the supernatants from infected RD cells for 2 h. Cells were washed with phosphate buffer saline (PBS) and cultured with 2% FBS in DMEM. At day three post-infection, the plates were examined for the lowest dilution at which 50% of the wells showed the cytopathic effect using the Reed-Muench method [36].

### 2.9. Plaque Assay

The supernatant collected from infected RD cells was added to a 12-well plate containing Vero cells at about 90% confluence. After 2 h incubation, the cells were washed with PBS; then, 1 mL of plaque medium supplemented with 1% CMC-nZVI was added to each well immediately. The plate was incubated at 37 °C for three days. After incubation, the plaque medium was removed, and cells were fixed and stained with 4% formaldehyde and 0.5% crystal violet.

### 2.10. Coimmunoprecipitation (Co-IP)

HEK293T cells were lysed using lysis buffer at 4 °C for 1 h, and anti-GFP antibody was added to the lysates for overnight incubation at 4 °C. Protein-G (#14001578-EE) (GE Healthcare) was added to lysates and incubated for an additional 2 h. Immunocomplexes were washed thrice with cell lysis buffer, and subsequently boiled in 2× loading buffer for 10 min. Proteins were detected by Western blotting, as described. 

### 2.11. Confocal Microscopy

Plasmid-transfected HEK239T cells were fixed with 4% paraformaldehyde for 15 min at room temperature, permeabilized with 0.2% Triton-X100 for 5 min, and blocked with 5% BSA for 1 h. Murine monoclonal GFP and HA antibody were added to cells for overnight incubation at 4°C, and then FITC-conjugated anti-mouse and dylight 649-conjugated antirabbit secondary antibodies were incubated with cells for 1 h. Finally, cells were stained with DAPI for 5 min, and confocal imaging was performed using Fluo View FV1000 (Olympus, Tokyo, Japan).

### 2.12. Oligonucleotide Microarray Assay

RD cells were mock-infected or infected with EV71 (MOI = 0.1) for 12 h. The cells were then extracted with TRIzol reagent (Invitrogen); two samples were obtained for the analysis of the expression array (Human V1.0 whole-genome oligonucleotide microarray) at the Bioassay Laboratory of CapitalBio Corporation. The sample preparation method number was AG-SPRA-01, and the detection method number was AG-D01A-01. The data analysis number was AG-D01C-01. The results were shown using Microsoft Excel (Table S1).

### 2.13. Statistical Analyses

All values are expressed as the mean ± SEM. Statistical analysis was performed using the t test for two groups or one-way ANOVA (GraphPad Prism5) for multiple groups. *p* values below 0.05 were considered statistically significant.

## 3. Results

### 3.1. ILF2 Represses EV71 Infection in RD Cells

**Figure 1:**
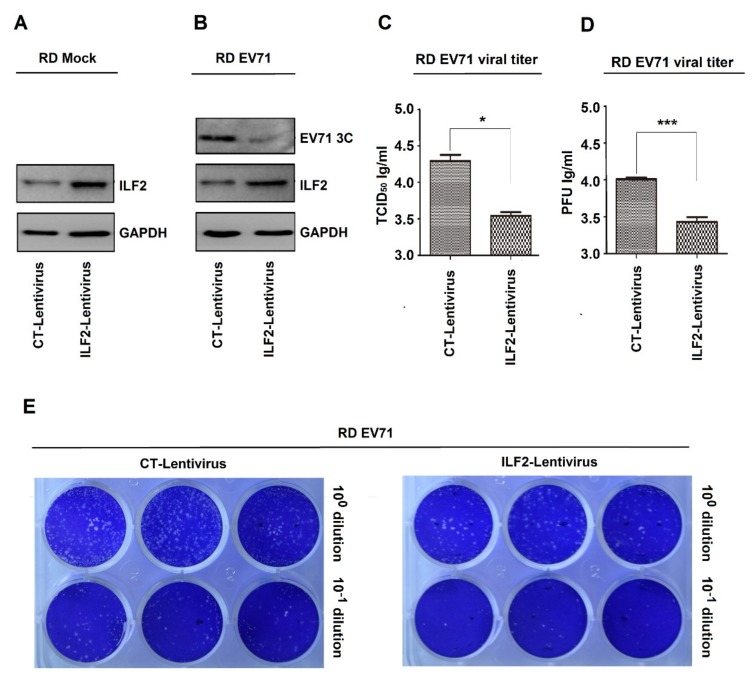
ILF2 represses EV71 infection in RD cells. (**A**) RD cells infected with the recombinant lentiviruses, ILF2 lentivirus expressing ILF2 and its control CT lentivirus, to generate two stable cell lines. ILF2 and GAPDH proteins expressed in the lysates of stable cell lines were detected by Western blot analysis. (**B**) The two stable cell lines infected with EV71 (MOI = 0.5) for 12 h. ILF2, EV71 3C, and GAPDH proteins expressed in the lysates of stable cell lines were detected by Western blot analysis. (**C**–**E**) The stable cell lines infected with EV71 (MOI = 0.5) for 12 h, and the supernatants of cell cultures collected and then inoculated into Vero cells. (**C**) The levels of EV71 TCID50 determined by TCID50 assays. (**D**) The levels of EV71 PFU determined by plaque assays. (**E**) Data presented in (**C**) and (**D**) visualized under a camera. * *p* < 0.05, and *** *p* < 0.0001.

The role of ILF2 in the regulation of EV71 infection was initially determined. Two recombinant lentiviruses, i.e., ILF2 lentivirus and its control CT lentivirus, were constructed based on the procedures described previously [37]. Human RD cells were infected with CT lentivirus and ILF2 lentivirus to generate two stable cell lines. Western blot analyses showed that a basal level of endogenous ILF2 was detected in CT-lentivirus cells, while a significantly higher level of ILF2 was produced in ILF2-lentivirus cells (Figure 1A), indicating that ILF2 is stably expressed in ILF2-lentivirus cells. Upon EV71 infection, EV71 3C was attenuated in ILF2-lentivirus cells as compared with CT-lentivirus cells (Figure 1B), demonstrating that ILF2 represses EV71 replication. In addition, the cells were infected with EV71, and the supernatants were collected for TCID50 assays. EV71 TCID50 was significantly downregulated in ILF2-lentivirus cells, as compared with CT-lentivirus cells (Figure 1C), indicating that ILF2 inhibits EV71 infection. Moreover, the cells were infected with EV71, and the supernatants were collected for plaque-formation assays. PFU was remarkedly attenuated in ILF2-lentivirus cells as compared with CT-lentivirus cells (Figure 1D,E), suggesting that ILF2 attenuates EV71 infection. Therefore, these data provide the first evidence that ILF2 represses EV71 infection.

### 3.2. ILF2 Expression is Attenuated in EV71-Infected Cells

**Figure 2:**
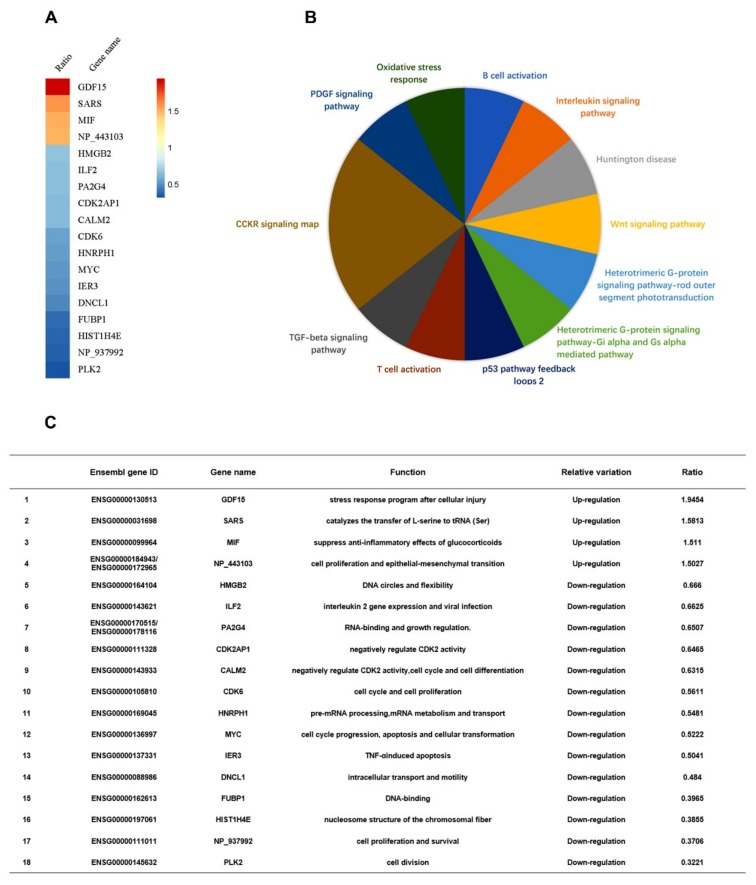
ILF2 expression is attenuated in EV71-infected cells. (**A**) The levels of differentially-expressed gene mRNAs analyzed by microarrays of RD cells infected with or without EV71 (MOI = 0.5) for 12 h. Z-score analyses were used to choose the significant differentially-expressed genes. The mRNAs of genes with more than 1.5-fold variations of expression levels were defined as differentially-expressed genes. Intensity ratio (experimental group/control group) of genes was calculated and visualized in R software (version 3.4.4) with Pheatmap Packages (version 1.0.8). (**B**) Differentially-expressed genes imported to the Protein Analysis Through Evolutionary Relationships Classification System to conduct pathway analysis. (**C**) Summary of Ensembl gene IDs, gene names, functions, and relative variations of the differentially-expressed genes.

Next, we determined whether EV71 plays any role in the regulation of ILF2. Differential mRNA expression of intracellular genes in two samples, i.e., one EV71-infected RD cells and one mock-infected RD cells, was initially evaluated through microarray analyses (Table S1). Z-score analyses of the microarray data showed that in EV71-infected cells, high mobility group box 2, ILF2, proliferation-associated 2G4, cyclin-dependent kinase 2-associated protein 1, calmodulin 2, cyclin-dependent kinase 6, heterogeneous nuclear ribonucleoprotein H1, Myc proto-oncogene protein, immediate early response 3, dynein light chain LC8-type 1, far upstream element-binding protein 1, histone cluster 1 H4 family member e, and polo-like kinase 2 were downregulated, while the growth differentiation factor 15, seryl-tRNA synthetase, and macrophage migration inhibitory factor were upregulated (Figure 2A). Protein Analysis Through Evolutionary Relationships revealed that cholecystokinin receptor pathway, platelet-derived growth factor signaling, oxidative stress response, B-cell activation, interleukin signaling, Huntington disease, Wnt pathway, heterotrimeric G-protein signaling pathway-rod outer segment, heterotrimeric G-protein signaling pathway-Gi alpha subunit, Gi alpha-mediated pathway, p53 pathway positive and negative feedback loops 2, T-cell activation, and transforming growth factor-β signaling pathway were involved (Figure 2B). The basic functions of the proteins were summarized (see Figure 2C). Therefore, microarray analyses showed that ILF2 expression is attenuated in EV71-infected cells. 

### 3.3. EV71 Represses ILF2 mRNA Expression and Protein Production

**Figure 3:**
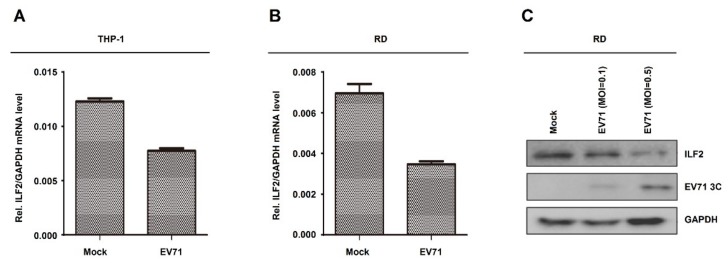
EV71 represses ILF2 mRNA expression and protein production. (**A**) THP-1 cells differentiated into macrophages upon treatment with 12-o-tetradecanoylphorbol-13-acetate (TPA). TPA-differentiated THP-1 macrophages were infected with or without EV71 (MOI = 5) for 36 h. The mRNA level of ILF2 gene was quantified by real-time PCR. (**B**) RD cells infected with or without EV71 (MOI = 1) for 12 h. The mRNA level of ILF2 gene was quantified by real-time PCR. (**C**) RD cells infected with or without EV71 (MOI = 0.1 or 0.5) for 12 h. ILF2, EV71 3C, and GAPDH proteins expressed in the cell lysates were detected by Western blot analysis. Data shown are means ± SEMs.

Next, the role of EV71 in the regulation of ILF2 expression was determined in human leukemic monocytes (THP-1) cells and RD cells. THP-1 cells were differentiated into macrophages upon treatment with TPA as described previously [38,39]. ILF2 mRNA was downregulated upon EV71 infection in THP-1-differentiated macrophages (Figure 3A). Similarly, ILF2 mRNA was attenuated by EV71 infection in RD cells (Figure 3B). These results are consistent with the microarray data, and demonstrate that EV71 infection represses ILF2 mRNA expression. Moreover, ILF2 protein was downregulated and EV71 3C protein was produced in EV71-infected RD cells (Figure 3C), suggesting that EV71 attenuates ILF2 protein production. Taken together, we demonstrate that EV71 represses ILF2 mRNA expression and protein production.

### 3.4. EV71 2B Interacts and Colocalizes with ILF2 in the Cytoplasm

**Figure 4:**
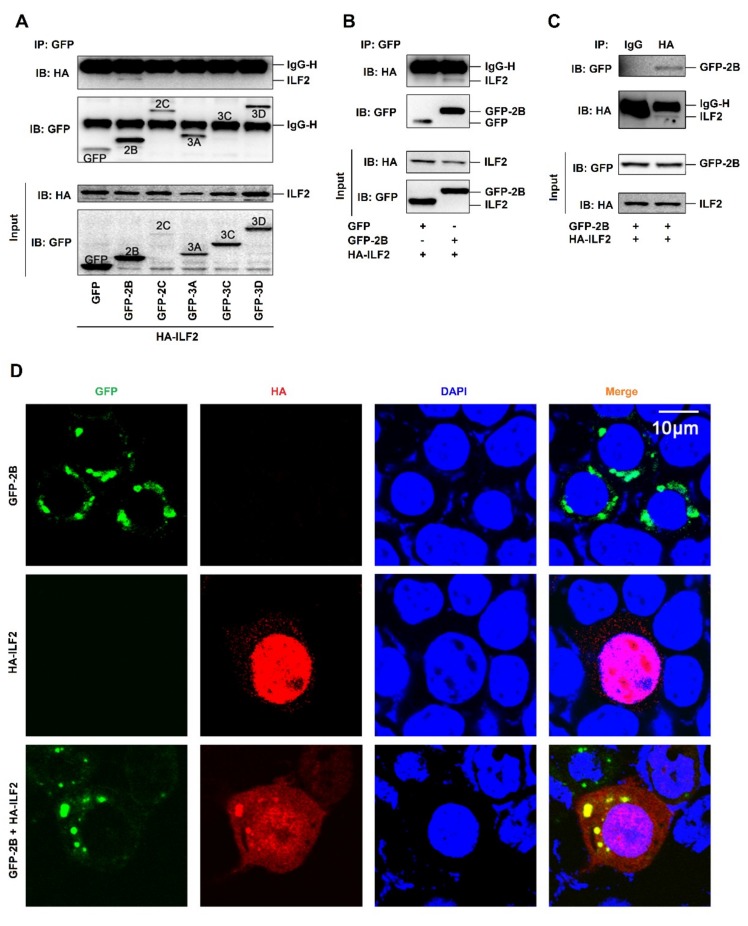
EV71 2B interacts and colocalizes with ILF2 in the cytoplasm. (**A**) Human embryonic kidney (HEK293T) cells co-transfected with plasmids encoding HA-ILF2 and each of EV71 nonstructure proteins, GFP-2B, GFP-2C, GFP-3A, GFP-3C, and GFP-3D. The GFP-tag, GFP-2B, GFP-2C, GFP-3A, GFP-3C, GFP-3D, and HA-ILF2 proteins were subjected to coimmunoprecipitation (Co-IP) assay with anti-GFP antibody or with IgG as a negative control. The levels of GFP-tag, GFP-2B, GFP-2C, GFP-3A, GFP-3C, GFP-3D, and HA-ILF2 proteins were detected by Western blot analyses using anti-GFP antibody or anti-HA antibody, as indicated. (**B** and **C**) HEK293T cells co-transfected with pHA-ILF2 and pGFP-tag or pGFP-2B. The GFP-tag, GFP-2B, and HA-ILF2 proteins were subjected to Co-IP assay with anti-GFP antibody (**B**). The GFP-tag, GFP-2B, and HA-ILF2 proteins were subjected to Co-IP assay with anti-HA antibody or with IgG as a negative control (**C**). The levels of GFP-tag, GFP-2B, and HA-ILF2 proteins were measured by Western blot using anti-GFP antibody or anti-HA antibody, as indicated. (**D**) HEK293T cells transfected with pGFP-2B alone or pHA-ILF2 alone or co-transfected with pGFP-2B and pHA-ILF2 together. The localization and distribution of EV71 2B protein (green), ILF2 protein (red), and the nuclei (blue) were visualized under a laser scanning confocal microscope.

To further evaluate the effect of EV71 on the regulation of ILF2, the interactions among EV71 proteins and ILF2 were determined. Human embryonic kidney (HEK293T) cells were co-transfected with plasmids encoding HA-ILF2 and each of the EV71 nonstructure proteins, i.e., GFP-2B, GFP-2C, GFP-3A, GFP-3C, and GFP-3D. Coimmunoprecipitation (Co-IP) results showed that EV71 2B interacted with ILF2, while other EV71 proteins failed to interact with ILF2 (Figure 4A). In addition, 2B interacted with ILF2 (Figure 4B), and ILF2 associated with 2B (Figure 4C) in HEK293T cells, confirming the interaction between 2B and ILF2. Since 2B distributes in the cytoplasm [8] while ILF2 mainly locates in the nucleus [33], we next determined whether 2B colocalizes with ILF2 in HEK293T cells. A laser scanning confocal microscope showed that 2B (green) alone was mainly localized in the cytoplasm (Figure 4D, top panels), and that ILF2 (red) alone was strongly distributed in the nucleus (Figure 4D, middle panels); however, in the presence of both proteins, 2B and the majority of ILF2 were colocalized in the cytoplasm to form spots (Figure 4D, bottom panels). These results suggest that 2B is colocalized with ILF2 in the cytoplasm by attenuating ILF2 nucleus translocation.

## 4. Discussion

EV71 infection causes HFMD, lymphopenia, herpetic pharyngitis, and neurological diseases, including brain stemencephalitis, aseptic meningitis, acute flaccid paralysis, poliomyelitis-like paralysis, and acute flaccid paralysis [4,40,41,42]. Therefore, it is important to study the mechanisms by which the host regulates EV71 infection and the virus antagonizes host-mediated antiviral effects. In this study, we initially show that ILF2 downregulates EV71 TCID50 and attenuates EV71 PFU, thereby repressing EV71 infection. Although this result is similar to previous reports showing that ILF2 regulates the replication of other viruses [28,29,30,31,32,33], this is the first study providing evidence that ILF2 represses EV71 infection. And although ILF2 plays critical roles in the repression of viral infection, the mechanism by which virus antagonizes ILF2-mediated antiviral effects was unknown until this study. ILF2 mRNA expression and protein production are repressed upon EV71 infection in THP-1-differentiated macrophages and RD cells. In addition, EV71 2B interacts with ILF2 to facilitate ILF2 translocation from the nucleus to the cytoplasm and colocalize with 2B in the cytoplasm. 

EV71 2B is involved in viral RNA replication [7], activates the mitochondrial cell death pathway [8], and enhances viral release [9]. This study demonstrates a new role of 2B in the regulation of ILF2. Previous study reported that 2B attenuates RIG-I-mediated antiviral effects through the repression of RIG-I expression [10]. We demonstrate that 2B antagonizes ILF2-mediated antiviral effects by repressing ILF2 expression and interacting with ILF2 to promote its translocation from the nucleus to the cytoplasm, thereby altering ILF2 function. Since ILF2 is a transcription factor that mainly locates and functions in the nucleus [33], EV71 2B-mediated ILF2 translocation from the nucleus to the cytoplasm must lead to the inhibition of ILF2 function.

In conclusion, we present a distinct mechanism by which EV71 antagonizes ILF2-mediated antiviral effects by inhibiting ILF2 expression and promoting ILF2 translocation from the nucleus to the cytoplasm through 2B protein.

## Supplementary Materials

The following are available online at https://www.mdpi.com/1999-4915/12/1/22/s1, Table S1: The data of microarray analyses of genes differentially expressed in Mock-infected and EV71-infected cells.
